# Vitamin D status and its association with insulin resistance among type 2 diabetics: A case -control study in Ghana

**DOI:** 10.1371/journal.pone.0175388

**Published:** 2017-04-19

**Authors:** Linda Ahenkorah Fondjo, William K. B. A. Owiredu, Samuel Asamoah Sakyi, Edwin Ferguson Laing, Michael Acquaye Adotey-Kwofie, Enoch Odame Antoh, Eric Detoh

**Affiliations:** 1 Department of Molecular Medicine, School of Medical Sciences, College of Health Sciences, Kwame Nkrumah University of Science and Technology, Kumasi, Ghana; 2 Nkawie Government Hospital, Kumasi, Ghana; Shanghai Diabetes Institute, CHINA

## Abstract

**Background:**

Vitamin D plays a major role in physiological processes that modulate mineral metabolism and immune function with probable link to several chronic and infectious conditions. Emerging data suggests a possible influence of vitamin D on glucose homeostasis. This study sought to provide preliminary information on vitamin D status among Ghanaian type 2 diabetics and assessed its association with glucose homeostasis.

**Methods:**

In a case control study, 118 clinically diagnosed Type 2 Diabetes Mellitus (T2DM) patients attending Diabetic Clinic at the Nkawie Government Hospital were enrolled between October and December 2015. Hundred healthy non-diabetics living in Nkawie district were selected as controls. Structured questionnaires were administered to obtain socio-demographic data. Venous blood samples were taken from both cases and controls to estimate their FBG, Lipid profile spectrophotometrically and IPTH, 25OHD by ELISA. Statistical analyses were performed using SPSS v20.0 Statistics.

**Results:**

The average age of the study participants was 58.81years for cases and 57.79year for controls. There was vitamin D deficiency of 92.4% among T2DM cases and 60.2% among the non diabetic controls. Vitamin D deficiency did not significantly associate with HOMA-**β** [T2DM: r^2^ = 0.0209, p = 0.1338 and Control: r^2^ = 0.0213, p = 0.2703] and HOMA-IR [T2DM: r^2^ = 0.0233, p = 0.1132 and Control: r^2^ = 0.0214, p = 0.2690] in both the controls and the cases.

**Conclusion:**

Vitamin D deficiency is prevalent in both T2DM and non-diabetics. There is no association between vitamin D deficiency and insulin resistance or beta cell function in our study population. Vitamin D supplementation among type 2 diabetics is recommended.

## Introduction

The vitamin D–endocrine system plays a major role in physiological processes that modulate mineral metabolism and immune function with probable link to several chronic and infectious conditions [1, 2]. Many recent studies have documented hypovitaminosis D to be associated with many disease conditions such as cancers, cardiovascular disease and diabetes mellitus [3]. In addition, some studies have reported a possible influence of vitamin D on glucose homeostasis [4, 5].

Currently in Ghana, type 2 diabetes mellitus (T2DM) affects at least 6% of urban adults’ population with 23% being overweight [6]. Epidemiological data indicate interactions between acculturation, urbanization and genetic disposition are involved in T2DM among Ghanaians [7]. However, the increasing incidence of T2DM is largely attributable to changes in lifestyle including modifications in diet and reduced physical activity as well as obesity.

It has been widely recommended that lifestyle modification, particularly weight loss with increased physical activity delays the progression of diabetes [8]. However, because weight loss is difficult to be maintained, it has become necessary to identify modifiable determinants for primary prevention of diabetes mellitus. In some recent studies, vitamin D supplementation have been identified as a risk modifier for T2DM, as it improves insulin secretion and reduces insulin resistance in T2DM and non-diabetic subjects [5, 9, 10]. Accumulating evidence from human and animal model studies have linked vitamin D status to insulin secretion and insulin resistance, as both vitamin D receptor and 1- **α-**hydroxylase are present in the pancreatic **β** cells [11–13]. Furthermore, it has been reported that low concentrations of vitamin D is associated with impaired insulin sensitivity; whereas replacement with vitamin D in the deficient state shows improved insulin sensitivity [5, 13, 14]. Similarly, vitamin D receptor knockout or vitamin D deficiency impairs glucose induced insulin secretion whereas insulin secretory response improves after vitamin D supplementation in both animals and humans [13, 15, 16].

Vitamin D status is influenced by skin exposure to ultraviolet B radiation (UVB) from the sun and Ghana is a tropical country with abundance of sunshine. It is therefore assumed that the continuous skin exposure to the sun is adequate for the body to synthesize the minimum recommended vitamin D the body needs, but vitamin D status and supply is not only influenced by sunshine exposure but also by diet, lifestyle and underlying health conditions [17].

In the face of the high T2DM among Ghanaians, we hypothesize that Ghanaian type 2 diabetics may present with high prevalence of vitamin D deficiency and a positive association with glucose homeostasis. This study therefore, sought to provide preliminary information on vitamin D status among Ghanaian type 2 diabetics and assessed its association with glucose homeostasis.

## Materials and methods

### Study design

This case-control study was conducted at the Diabetic Clinic of the Nkawie Government Hospital in Kumasi, Ghana, between the period of October and December 2015.

### Study area

The study was conducted at Atwima Nwabiagya District of the Ashanti Region. The District lies approximately on latitude latitude 6° 32’N and 6° 75’N and between longitude 1° 45’ and 2° 00’ West. It is one of the 30 political and administrative districts in the Ashanti Region. It covers an estimated area of 294.84 sq km. The population of the Atwima Nwabiagya District is 149,025 and the District capital is Nkawie (District Profile, 2009; Ghana Statistical Service, 2010 Population and Housing Census).

### Ethical considerations

Ethical approval for this study was obtained from the Committee on Human Research, Publication and Ethics (CHRPE) at the School of Medical Sciences, Kwame Nkrumah University of Science and technology (CHPRE /AP/ 355/15) as well as the Institutional review board of the Hospital. All participants gave their written informed consent after the aim and objectives of the study had been explained to them.

### Study population

A total of 118 clinically diagnosed type 2 diabetic patients attending Diabetic Clinic at the Nkawie Government Hospital for the past six months and had had their clinical history taken were enrolled in the study. Healthy 100 non-diabetics living in the Nkawie district were independently selected as controls for the study. Structured questionnaires were administered to them to obtain demographic and other socioeconomic data and venous blood samples were also taken from all the study participants.

### Inclusion and exclusion criteria

Subjects who satisfied the following criteria were included in the study: clinically diagnosed type 2 diabetic mellitus patients, 25 years and above, of more than six months duration were selected as subjects whilst non diabetics with Fasting Blood Glucose (FBG) less than 6.4 mmol/L were used as controls. Pregnant women, patients with history or clinical features suggesting chronic liver diseases, chronic illness such as hypertension, renal disease, patients with medication that could affect glucose or lipid metabolism and intake of vitamin D or calcium supplements were all excluded from the study.

### Anthropometric measurements

The weight of selected subjects and controls were measured in light clothing without shoes using an analogue weighing scale (Seca, Hamburg, Deutschland) (to the nearest 0.1 kg). Height was measured without shoes using a stadiometer (Seca, Hamburg, Deutschland) (to the nearest 0.1 cm). Waist circumference (WC) and hip circumference (HC) was measured (to the nearest 0.1 cm). Body Mass Index (BMI) was calculated using the equation; BMI = weight/height^2^ (kg/m^2^). Visceral adiposity index (VAI) and Body adiposity index (BAI) were calculated using the formulae below:

VAI for:

$$Males=\left(\frac{WC}{39.68+\left(1.88\times BMI\right)}\right)\times \left(\frac{TG}{1.03}\right)\times \left(\frac{1.31}{HDL-C}\right)$$

$$Females=\left(\frac{WC}{36.58+\left(1.89\times BMI\right)}\right)\times \left(\frac{TG}{0.81}\right)\times \left(\frac{1.52}{HDL-C}\right)$$

$$BAI=\frac{waist\;circumference}{height{\left(m\right)}^{1.5}}-18$$

### Sample collection and preparation

Ten (10) milliliters of blood were collected from the ante cubital vein of each respondent between the hours of 8am to 11am after an overnight fast for the biochemical assays. Two (2) milliliters of blood was dispensed into tubes containing fluoride oxalate and 8ml of blood was dispensed into gel separator tubes. The tubes were placed in a centrifuge and spun at 3000 rpm for 10 minutes to obtain the plasma and serum. Plasma glucose was measured immediately and the serum and plasma for the measurement of other biochemical variables were stored at -20°C until analysis.

### Biochemical assays

Biochemical reagents for fasting blood glucose, total cholesterol, HDL-cholesterol, triglycerides, and LDL-cholesterol were purchased from Teco Diagnostics Co. LTD, USA and were used to analyze the samples spectrophotometrically using the UV 2005 spectrophotometer (J.P SELECTA SAU, Barcelona, Spain). 25-Hydroxy Vitamin D [25(OH)D], Intact Parathyroid Hormone (IPTH), and Human Insulin were purchased from Green Stone Swiss Co. LTD, China. These reagents were used to determine the 25(OH)D, IPTH and Insulin levels of all the study participants based on the principle of solid phase enzyme linked immunosorbent assay (ELISA) according to the manufacturer’s instructions. Subsequently, their respective absorbances were measured spectrophotometrically using Mindray (MR-96A, USA) ELISA plate reader. Serum levels of 25(OH)D were stratified into optimal, severe deficiency, mild to moderate, increased risk and toxic levels as follows: 20-50ng/ml, < 10ng/ml, 10-19ng/ml, 51-80ng/ml and >80ng/ml. Subsequently, all 25(OH)D values of <20 ng/ml were considered as vitamin D deficient and **≥** 20 ng/ml as non-deficient [18]

The Homeostasis Model of Assessment-Insulin Resistance Index (HOMA-IR) and Homeostatic Model of Assessment- Beta (HOMA– β) in mmol/L, were used as an index of insulin resistance and were calculated from the formulae below:

$$HOMA-IR=\frac{\left(Fasting\;Insulin\times \;Fasting\;Blood\;Sugar\right)}{22.5}$$

HOMA- β was calculated in mmol/L as:

$$\text{HOMA}-\;\beta =\frac{20\;\times \;Insulin}{Glucose-3.5}\;\%$$

### Statistical analysis

All data were presented as frequency (percentages) and Means ± SD and Median (interquartile range) where appropriate. Unpaired *t-*test was used to compare between two means of all parametric continuous variables. Mann-Whitney U test was used to compare between two means of all non-parametric continuous variables. The Chi-square test statistic was used to test association between of categorical variables. Unconditional logistic regression analysis was performed to determine the odds of Vitamin D status in predicting T2DM. Linear regression analysis was also performed to test association between biochemical parameters. A p-value < 0.05 was considered statistically significant. All statistical analyses were performed using IBM SPSS 20.0 Statistics.

## Results

The socio-demographic characteristics of study participants are shown in Table 1. The mean age of the diabetics (58.81years) was not significantly different from non-diabetic controls (57.79year) (p = 0.5465). There were more females than male participants (169 vs 47). Higher proportion of T2DM patients were married (82.2%), had completed primary education (75.4%) and were Christians (94.9%). None of the participants had a history of smoking. Out of 118 diabetics, 9 representing 7.6% had history of alcoholic beverage intake. All the respondents were resident in the study area and majority of them were subsistent farmers with regular exposure to sunshine especially during working hours.

**Table 1:** Socio-demographic and clinical characteristics of study participants.

Variables	NON-T2DM(n = 98)	T2DM (n = 118)	*(X2*,*df) p-value*
**Gender**			(0.05, 1) 0.8692
Male	22(22.4%)	25(21.2%)	
Female	76(77.6%)	93(78.8%)	
**Marital status**			**(17.40, 3) 0.0006**
Single	3(3.1%)	3(2.5%)	
Married	62(63.3%)	97(82.2%)	
Divorced	11(11.2%)	0(0.0)	
Widow	22(22.4%)	18(15.3%)	
**Highest level of education**			**(7.83, 3) 0.0498**
Unschooled	26(26.5%)	20(16.9%)	
Basic	62(63.3%)	89(75.4%)	
Secondary	0(0.0)	3(2.5%)	
Tertiary	10(10.2%)	6(5.1%)	
**Type of religion**			**(8.62, 2) 0.0134**
Christianity	90(91.8%)	112(94.9%)	
Islamic	2(2.0%)	6(5.1%)	
Others	6(6.1%)	0(0.0)	
**Alcoholic beverage intake**			(0.82, 1) 0.3638
Yes	11(11.2%)	9(7.6%)	
No	87(88.8%)	109(92.4%)	
**History of smoking**			(2.43, 1) 0.119
Yes	2(2.0%)	0(0.0)	
No	96(98.0%)	118(100.0%)	

P-values of significant variables are in bold print X^2^,df: Chi-square test, degree of freedom. p<0.05 was considered statistically significant different between categorical variable among T2DM and non-T2DM: T2DM: type 2 diabetes mellitus

Table 2 shows anthropometric and biochemical profile of study participants. Participants with T2DM had elevated weight (p = 0.0031), BMI (p = 0.001), FBG (<0.0001), Insulin (p = 0.0088), intact PTH (p = 0.0349), HOMA-IR (p< 0.0001), TG (p = 0.0113), LDL-c (0.0184), TC (p = 0.1967) and BAI (p = 0.0046) and wider WC (p = 0.0004) and HC (p = 0.0012) with corresponding decrease HDL-C (p = 0.0008), 25(OH)D (p< 0.0001), HOMA- β (p< 0.0001) compared to non-T2DM participants.

**Table 2:** Anthropometric and biochemical profile of study participants.

Parameters	NON-T2DM (n = 98)	T2DM (n = 118)	p-value
**Age**	57.79 ± 1.49	58.81 ± 0.90	0.5465
**Anthropometrics**			
Weight (kg)	60.93 ± 1.48	66.57 ± 1.19	**0.0031**
Height (m)	1.599 ± 0.01	1.599 ± 0.01	0.9394
BMI (kg/m^2^)	23.73 ± 0.51	26.05 ± 0.47	**0.001**
WC (cm)	87.73 ± 0.91	92.33 ± 0.89	**0.0004**
HC (cm)	96.84 ± 0.86	101.1 ± 0.94	**0.0012**
WHR	0.91 ± 0.01	0.89 ± 0.01	0.4413
VAI	0.11 ± 0.02	0.10 ± 0.02	0.8060
BAI	30.04 ± 0.48	32.15 ± 0.54	**0.0046**
**Biochemical profile**			
TG (mg/dl)	120.30 ± 8.51	141.5 ± 10.98	0.1413
TC (mg/dl)	186.44 ± 0.14	196.86 ± 0.15	0.1967
HDL-c (mg/dl)	41.92 ± 1.93	33.61 ± 1.56	**0.0008**
LDL-c (mg/dl)	122.8 ± 6.18	137.5 ± 5.57	**0.0184**
FBG(mmol/L)	5.32 ± 0.09	11.03 ± 0.34	**< 0.0001**
25(OH)D (ng/ml)	12.56(2.62–32.77)	2.45(1.93–8.96)	**< 0.0001**
Insulin (mU/L)	0.65(0.53–0.87)	0.95(0.51–0.85)	**0.0088**
IPTH (ng/L)	4.55(3.61–6.62)	5.19(3.97–7.57)	**0.0349**
HOMA-IR	0.16(0.13–0.23)	0.32(0.23–0.41)	**< 0.0001**
HOMA- β (%)	6.66(4.09–10.10)	1.99(1.54–2.58)	**< 0.0001**

Values are presented as mean ± SD and Median (interquartile range), P-values of significant variables are in bold prints. P<0.05 was considered statistically significant difference. Unpaired t-test was performed to compare between parametric variable presented as mean±SD. Mann Whitney U test was performed to compare between non-parametric variable presented as median (interquartile ranges). BMI: Body mass index; WC: Waist circumference; HC: Hip circumference; WHR: Waist to hip ratio; VAI: Visceral adiposity index; BAI: Body adiposity index: TG: Triglyceride: TC: Total cholesterol; HDL-c: High density lipoprotein cholesterol: LDL-c: Low density lipoprotein cholesterol: FBG: Fasting blood glucose; 25(0H)D: 25-hydroxy vitamin D: IPTH: Intact parathyroid hormone: HOMA-IR: Homeostatic model assessment –insulin resistance: HOMA-β: Homeostatic model assessment-beta.

Table 3 shows baseline demographic, anthropometrics and biochemical indices of T2DM cases and control subjects stratified by vitamin D status. With the exception of WHR that was significantly lower among vitamin D deficient T2DM compared to vitamin D sufficient T2DM participants (p = 0.0051), other anthropometric indices such as BMI, WC, HC, WHR, VAI and BAI showed no significant difference (p>0.05). Also levels of FBG (p<0.0001), insulin (p = 0.0057) and HOMA-IR (p<0.0001) were significantly lower in vitamin D deficient T2DM compared to vitamin D sufficient participants though TG, TC, LDL, HDL, IPTH and HOMA-β levels showed no significant difference. For non-diabetic (NON-T2DM) controls, participants with vitamin D deficiency were significantly older (p<0.0001), with elevated TG (p = 0.0042) and FBS (p<0.0001) but lower HOMA- β (p<0.0085) compared to those with vitamin D sufficiency.

**Table 3:** Baseline demographic, anthropometrics and biochemical indices of T2DM cases and control subjects stratified by vitamin D status.

Parameters	T2DM CASE (n = 118)		*p-value*	NON-T2DM CONTROL (n = 98)		*p-value*
	Deficient (<20 ng/ml)	Sufficient (≥20 ng/ml)		Deficient (<20 ng/ml)	Sufficient (≥20 ng/ml)	
	(n = 109)	(n = 9)		(n = 59)	(n = 39)	
Age	58.93 ± 0.96	57.33 ± 2.59	0.6439	62.85 ± 1.81	50.13 ± 2.05	**< 0.0001**
**Anthropometrics**						
BMI (kg/m^2^)	26.17 ± 0.49	24.60 ± 1.11	0.3751	23.15 ± 0.65	24.62 ± 0.81	0.1585
WC(cm)	92.22 ± 0.95	93.67 ± 1.97	0.668	87.39 ± 1.24	88.26 ± 1.35	0.6452
HC(cm)	101.4 ± 0.99	97.33 ± 2.60	0.2507	96.00 ± 1.12	98.10 ± 1.35	0.2342
WHR	0.91 ± 0.01	0.96 ± 0.01	**0.0051**	0.91 ± 0.01	0.90 ± 0.01	0.4325
VAI	0.11 ± 0.01	0.16 ± 0.03	0.1922	0.10 ± 0.01	0.12 ± 0.02	0.5749
BAI	32.26 ± 0.58	30.78 ± 1.21	0.4675	29.79 ± 0.57	30.42 ± 0.83	0.5252
**Biochemical indices**						
TG (mg/dl)	141.1 ± 11.71	146.7 ± 26.61	0.893	139.9 ± 12.20	90.76 ± 9.10	**0.0042**
TC (mg/dl)	199.95 ± 0.16	161.73 ± 0.20	0.0812	186.82 ± 0.18	186.05 ± 0.21	0.9575
HDL-c (mg/dl)	34.23 ± 1.61	26.10 ± 5.97	0.1677	43.64 ± 2.79	39.32 ± 2.34	0.2755
LDL-c (mg/dl)	140.0 ± 5.91	106.3 ± 11.08	0.108	114.1 ± 7.59	135.9 ± 10.22	0.0852
FBG (mmol/L)	10.60 ± 0.27	16.23 ± 2.68	**< 0.0001**	6.05 ± 0.10	5.328 ± 0.09	**< 0.0001**
Insulin(mU/L)	0.63(0.49–0.85)	0.81(0.74–11.58)	**0.0057**	0.66(0.53–0.88)	0.63(0.53–0.87)	0.7767
IPTH (ng/L)	4.39(3.51–6.59)	4.85(3.89–284)	0.1118	6.06(3.97–7.96)	4.87(3.96–6.79)	0.419
HOMA-IR	0.29(0.22–0.39)	0.93(0.49–3.76)	**< 0.0001**	0.18(0.14–0.23)	0.15(0.12–0.24)	0.103
HOMA-**β (%)**	2.09(1.59–2.57)	1.31(0.71–43.7)	0.2729	5.73(3.64–7.64)	7.26(5.29–12.23)	**0.0085**

Values are presented as mean ± SD and Median (interquartile range), P-values of significant variables are in bold prints. P<0.05 was considered statistically significant difference. Unpaired t-test was performed to compare between parametric variable presented as mean±SD. Mann Whitney U test was performed to compare between non-parametric variable presented as median (interquartile ranges). BMI: Body mass index; WC: Waist circumference; HC: Hip circumference; WHR: Waist to hip ratio; VAI: Visceral adiposity index; BAI: Body adiposity index: TG: Triglyceride: TC: Total cholesterol; HDL-c: High density lipoprotein cholesterol: LDL-c: Low density lipoprotein cholesterol: FBG: Fasting blood glucose; 25(0H)D: 25-hydroxy vitamin D: IPTH: Intact parathyroid hormone: HOMA-IR: Homeostatic model assessment –insulin resistance: HOMA-β: Homeostatic model assessment-beta

The 25(OH) D status among T2DM and non-T2DM participants are shown in Table 4. The overall prevalence of 25(OH) D deficiency among T2DM participants was 92.4% (109/118) (severe deficiency and insufficiency were 78.0% (92/118) and 14.4% (17/115) respectively) while the prevalence among non-type 2 diabetic mellitus controls was 60.2% (59/98) (severe deficiency and insufficiency were 44.9% (44/98) and 15.3% (15/98) respectively). Logistic regression analysis indicated that participants with severe 25(OH) D deficiency were 22.3 times [aOR = 22.3, 95% CI (6.47 to 76.85), p<0.0001)] increase odds of being T2DM while those with mild to moderate deficiency were 12.09 times [aOR = 22.3, 95% CI (3.06 to 47.69), p = 0.0001)] increase odds of being T2DM. Increase 25 (OH) D level was not an independent risk factor of T2DM.

**Table 4:** Prevalence and vitamin D status among T2DM and non-T2DM participants.

	T2DM (n = 118)	NONT2DM (n = 98)	X^2^, df (p-value)	Age, BMI, gender adjusted aOR(95% CI)	*p-value*
**25(OH) D status**			41.60, 4 (< 0.0001)		
Severe deficiency	92 (78.0%)	44(44.9%)		22.30(6.47 to 76.85)	<0.0001
Mild to moderate deficiency	17(14.4%)	15(15.3%)		12.09(3.06 to 47.69)	0.0001
Optimal	3(2.5%)	32(32.7%)		1.0 (reference)	
Increase risk	3(2.5%)	6(6.1%)		5.33(0.86 to 33.01)	0.0891
Toxicity	3(2.5%)	1(1.0%)		0.03(0.00 to 0.41)	0.0082

X^2^, df; Chi-square, degree of freedom. aOR: adjusted odds ratio; CI: Confidence interval: model adjusted for age and gender. 25(OH) D: 25-hydroxyvitamin D

## Discussion

This study observed vitamin D deficiency of 92.4% among T2DM cases and 60.2% among the non-diabetic controls, with the diabetic cases showing significantly lower levels of vitamin D compared to the controls (Table 2). Ghana is a tropical country with abundant sunshine almost throughout the year, and it is assumed that its indigenes should have adequate vitamin D, however, this findings for the first time in Ghana has shown that vitamin D status and supply is not only influenced by sunshine exposure but also by other factors such as diet, lifestyle and underlying health conditions.

Sheth and coworkers [10] in a prospective cross sectional case control study among diabetics in India observed vitamin D deficiency in 91.4% and 93.0% of T2DM cases and control subjects respectively. In a cross sectional Iranian study by Taheri and colleagues [19], the prevalence of vitamin D deficiency was 83.3% in diabetic patients and 75.6% in healthy subjects. Another cross sectional study among rural and urban adult Indians, Harinarayan *et al*., [20] also observed 44% and 62% for rural and urban men respectively and 70% and 75% deficiency for rural and urban women respectively. An Iranian Multi–Center Osteoporosis Study (IOMS) showed that 75.1% of women and 72.1% of men in Iran have vitamin D deficiency [21]. Gupta [22] in a review conducted in India, reports of an endemic vitamin D deficiency of 70%- 100% across India. Spiro and Buttriss [23] in another review reported vitamin D deficiency of 2–30% in European adults. Vitamin D though prevalent across the globe shows wide variation in percentages.

Although the observed high deficiency rates have been attributed to low exposure to direct sunlight in some studies contrary in our study, however, exposure to sunshine, diet and health status play major contributing roles to vitamin D status. Although, there are little or no available studies investigating cutaneous synthesis of Vitamin D in indigenous African people who are predominantly deeply pigmented, it is thought however that the efficiency of vitamin D cutaneous synthesis depends on skin pigmentation and age [17, 24, 25]. Since Africans are generally deeply-pigmented, it presupposes that the high prevalence of hypovitaminosis D in this present study is due to deep skin pigmentation and advanced age as both groups had mean age of almost 60years. Similarly, in a Northern Indian cross sectional study, Marwaha *et al*., [26] have reported hypovitaminosis D in healthy Indians aged above 50 years.

Additionally, an overall poor nutrition and possibly low intake of dietary calcium could likewise account for the high deficiency levels as well the extremely low median levels of 25(OH)D from our study (Tables 1 and 2) as compared to other studies [5, 19]. Intake of dairy products is negligible in many African countries [17] and for that matter Ghana. Additionally, dairy products are considered an item of luxury to the socioeconomically challenged; as generally the respondents were low-income earners, due to the fact that most of them had attained only basic educational qualification and are peasant farmers who practice subsistent farming. These indeed can be a huge factor contributing to the high levels of hypovitaminosis D; due to the effect diet and calcium metabolism has on vitamin D synthesis.

Obesity is similarly associated with vitamin D deficiency due to excess adiposity, vitamin D is known to be sequestrated or stored in adipose tissues and due to the sizeable storage ability of the adipocytes, obese individuals tend to have lower circulating 25-hydroxyvitamin D concentrations [27, 28]. Evidently from this study, apart from WHR and VAI, all the other measures of obesity were significantly elevated in the diabetic study participants as compared to controls (Table 2). The diabetic cases, in whom vitamin D deficiency was higher percentage-wise, were in the overweight range (Table 2). Additionally, although only WHR was significantly higher in the deficient diabetics as compared to the non-deficient diabetics, generally the anthropometrics were above normal limits in the deficient diabetes (Table 3). Rodrguez-Rodrguez *et al*., [29] in a cross sectional study on Spanish women reported that overweight and obese women are at higher risk of vitamin D deficiency, principally due to excess adiposity. Taheri *et al*., [19], similarly, reported that serum level of 25(OH) D has an inverse relationship with BMI among adult population with and without T2DM in Iran. A study by Vimaleswaran and colleagues [28] have furthermore provided genetic evidence from across different populations, that higher BMI leads to lower vitamin D status among both men and women across different age groups, providing substantial evidence for the role of obesity as a pivotal risk factor for the development of hypovitaminosis D.

Additionally, LDL-c was significantly higher whereas HDL-c was significantly lower in the diabetics as compared to the controls (Table 2). Dyslipidaemia was observed in the vitamin D deficient T2DM as compared to the non-deficient T2DM controls although a borderline significant difference was observed in only total cholesterol (p = 0.0812) (Table 3). Dyslipidemia is a risk factor for cardiovascular diseases and recently some studies have demonstrated an association between hypovitaminosis D and dyslipidaemia [30, 31]. Similar to our study, Karhapaa and colleagues found that vitamin D deficiency was associated with high total cholesterol among Belgian men [30]. Martins and colleagues [32] whose study was conducted among non-Hispanic black and Mexican Americans aged 60 and above observed elevated total cholesterol levels with hypo 25-hydroxyvitamin D levels. Chaudhuri *et al*., [31] in a cross sectional study among asymptomatic Indians reported that, deficiency of 25(OH) D was associated with increased serum total cholesterol, triglycerides and LDL-C. Although, disarray in lipid metabolism is a hallmark of T2DM; obesity and hypovitaminosis D are important risk factors for T2DM [33–35]. This associated interplay between diet, age and obesity could all partly explain the higher deficiency rates observed in these study participants.

This study also observed higher levels of FBG among vitamin D sufficient diabetics as well as low levels (0.29, 0.93) of HOMA-IR in both vitamin D deficient and sufficient T2DM group (Table 3). This may be due to the fact that only a one point FBG sample was taken and may not represent the average FBG levels. Moreover, since glycated haemoglobin was not measured we cannot conclude if it represents actual levels of FBG and hence HOMA-IR among both groups.

**Fig 1:**
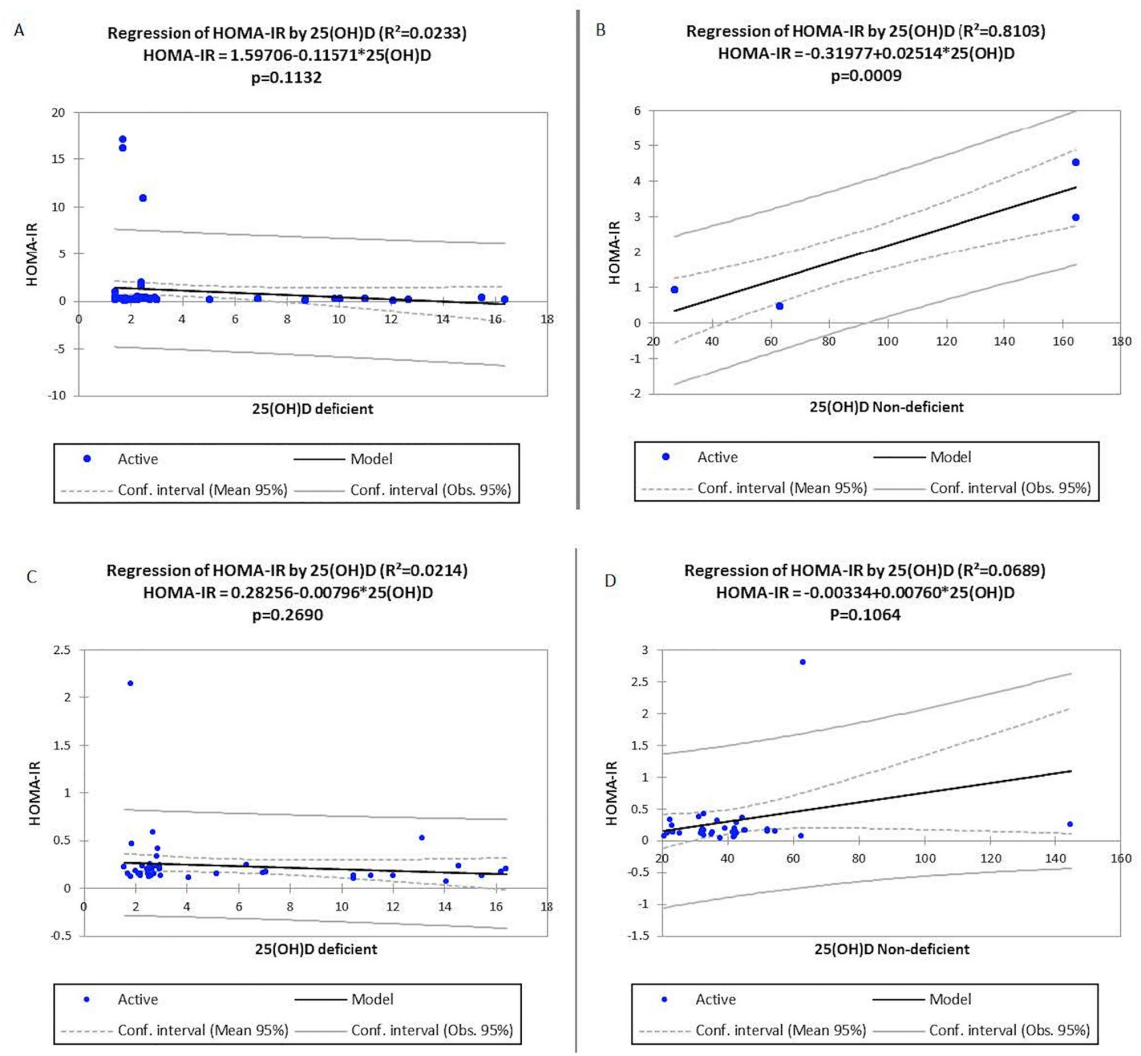
Linear regression between vitamin D levels and HOMA-IR in deficient and non-deficient T2DM and controls. Vitamin D deficiency did not have any statistically significant association with HOMA-IR in both the T2DM and control groups [T2DM: r^2^ = 0.0233, p = 0.1132 and Control: r^2^ = 0.0214, p = 0.2690] respectively Fig 1A and Fig 1C. Significant positive association was seen in T2DM with non-deficient vitamin D levels and HOMA-IR levels Fig 1B [T2DM: r^2^ = 0.8103, p = 0.0009] but no significant association was observed for Control: r^2^ = 0.0689, p = 0.1064] Fig 1D.

**Fig 2:**
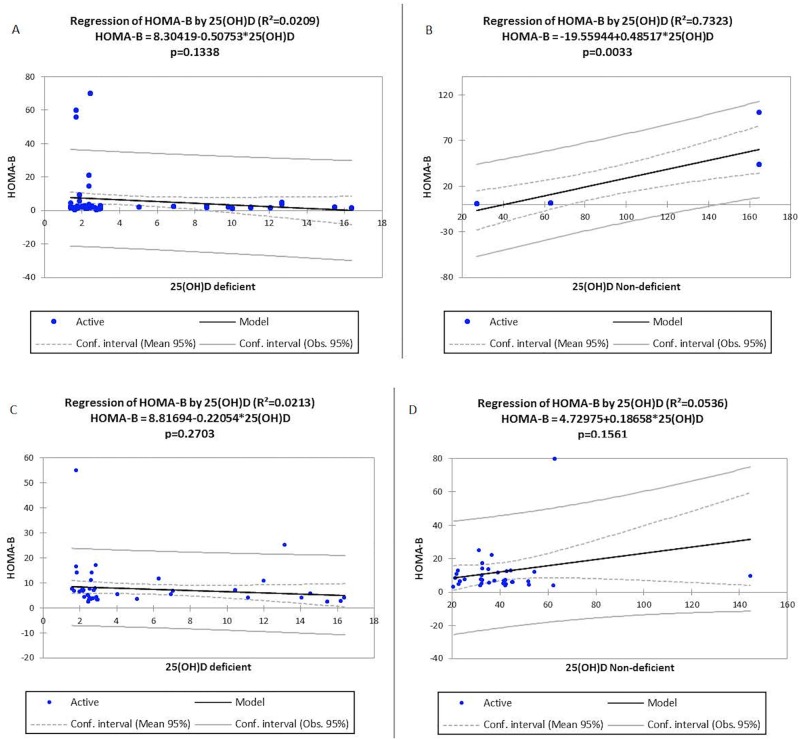
Linear regression between vitamin D levels and HOMA- β in deficient and non-deficient T2DM and controls. Vitamin D deficiency did not have any statistically significant association with HOMA- **β** in both groups [T2DM: r^2^ = 0.0209, p = 0.1338 and Control: r^2^ = 0.0213, p = 0.2703] respectively Fig 2A and 2C. Significant positive association was seen in T2DM with non-deficient vitamin D levels and HOMA- **β** levels [T2DM: r^2^ = 0.7323, p = 0.0033] Fig 2B but no significant association was observed for Control: r^2^ = 0.0536, p = 0.1561] Fig 2D.

Studies on the association between vitamin D deficiency and T2DM has been inconsistent while some have demonstrated a significant association between Vitamin D deficiency and Type 2 diabetes [4, 36–38] others have reported no association [10, 39]. Furthermore some studies have also suggested that vitamin D supplementation improves glucose homoestasis, insulin resistance and glucose control [5, 9]. This present study where only current levels of vitamin D were measured. A significant association between vitamin D deficiency and HOMA-IR as well as HOMA– β in both the control group and the diabetic cases was not observed. However, a positive association was observed between non-deficient T2DM and non-deficient controls (Figs 1 and 2). This finding is in part consistent with the findings of Sheth *et al*., and Luo *et al*., [10, 39]. Whose report indicated that an association between vitamin D deficient and HOMA-IR and glycemic control could not be confirmed. In addition, studies by Witham *et al*., [40] in a randomised, parallel group, placebo-controlled trial and Kampmann *et al*., [41] in a double-blind, randomized, placebo-controlled trial have also reported that improvement in vitamin D status had no effect on insulin resistance in patients T2DM. Elkassaby *et al*., [42] in an Australian randomized controlled trial of high dose vitamin D study conducted among recent-onset type 2 diabetes, reported that there was neither change in HbA1c nor beta cell function.

The lack of a significant association between hypovitaminosis D and insulin resistance as well as HOMA- β (Figs 1 and 2) in the diabetic study participants observed in our study, calls for further evaluation and a larger scale study, of the role vitamin D plays in T2DM, insulin resistance and overall glucose homeostasis. Additionally, in a study conducted by Inzucchi *et al*., [43] who observed improvement in insulin sensitivity of respondents on supplementation, their study indicated that the improvements were observed in study participants whose vitamin D levels had increased from 10 to 30ng/ml. Probably, lack of association observed in our study could be due to the fact that most of the respondents in this current study had severe vitamin D deficiency. Although both the control and cases presented with vitamin D deficiency, it is noteworthy that retrospective measurements of vitamin D were not assessed as such, we cannot conclude whether the deficiency of vitamin D precedes the development of T2DM in our respondents. But logistic regression analysis indicated that participants with severe 25(OH) D deficiency were 22.3 times [aOR = 22.3, 95% CI (6.47 to 76.85), p<0.0001)] at increased odds of being T2DM while those with mild to moderate deficiency were 12.09 times [aOR = 22.3, 95% CI (3.06 to 47.69), p = 0.0001)] (Table 4).

Remarkably, some of the respondents were within the increased risk and toxicity levels with regards to their vitamin D status (Table 4). Although information on all the current medication of the respondents were not documented, the observed high levels in those group of respondents could be due to their routine medications, as some thiazide diuretics and some hypertension medications are known to predispose one to increased vitamin D levels. The limitation of the current study is our inability to recall dietary and medication data from peri-urban population who were recruited, similarly, sun exposure habits of the respondents was not measured. This study did not also measure 25(OH)D levels before disease on-set as such a temporal relationship cannot be established by our study. However, these has no substantive effect on our main findings.

## Conclusion

Vitamin D deficiency is prevalent in both T2DM as well as the non-diabetic. Furthermore, there is no association between vitamin D deficiency and insulin resistance or beta cell function. However, we recommend vitamin D supplementation among type 2 diabetics.
